# Commentary: Chronic PD-1 Checkpoint Blockade Does Not Affect Cognition or Promote Tau Clearance in a Tauopathy Mouse Model

**DOI:** 10.3389/fnagi.2020.00135

**Published:** 2020-05-13

**Authors:** Kuti Baruch, Eti Yoles

**Affiliations:** ImmunoBrain Checkpoint Ltd., Ness Ziona, Israel

**Keywords:** Alzheimer's disease, immune checkpoints, immunotherapy, neurodegeneration, PD-1, PD-L1, chronic treatment

In a recent paper in *Frontiers in Aging Neuroscience* (Lin et al., 2020), the authors purported to examine treatment efficacy of Programmed cell death protein (PD)-1 antibody blockade in a tauopathy mouse model in a weekly administration regimen, which the authors refer to as “chronic,” supposedly based on the studies by Schwartz's team (Baruch et al., 2016; Rosenzweig et al., 2019). We wish to highlight several conceptual and technical critical issues in both study design and treatment approach, that preclude reaching any conclusion from this work. Accordingly, the title of the article and data interpretation are misleading. Furthermore, we wish to use this commentary and encourage the community to investigate Schwartz's therapeutic approach by using good scientific practice, and based on the suggested mechanism of action.

In the study by Lin et al. (2020), the authors used homozygous female JNPL3 mice, a mouse model of tauopathy, in which the same group has previously shown beneficial effects of active and passive tau immunization (Asuni et al., 2007; Boutajangout et al., 2011). In their previous studies, treatment started at the age of 2 months and outcome measurements (behavior and brain pathology) were tested at the ages of 4 to 8 months. In those studies, the authors emphasized that homozygous JNPL3 mice suffer from progressive sensorimotor abnormalities, but remain relatively healthy in these aspects at least until 8 months of age. Nevertheless, at 12 months of age these mice are severely impaired with hindlimb paralysis that prohibit any ability for behavioral testing (Asuni et al., 2007). The authors also described that the neurofibrillary pathology was much more extensive in females, up to the last time point tested−8 months of age.

Given the above-described previous reports by this team, it is surprising that the current study (Lin et al., 2020) is based upon results from an experiment performed using 22 female JNPL3 mice at the advanced age of 10–11 months, much older than previously used and at which according to the authors—the female mice suffer from severe motor disability. This cohort was divided into two groups, and tested for behavior and brain pathology, at 13–14 and 14–15 months of age, respectively. The authors apparently justified the use of such an aged cohort by claiming that there was a “shift” in their colony, and therefore mice could be tested at a more advanced age (EM Sigurdsson, “personal observation”; Methods section, Lin et al., 2020). Yet, no quantitative parameters were presented, neither in the present study nor in any of their previous publications to support this claim, and no data were shown using the authors' own tau therapy approach to validate testing of the mice at this old age. Rather, in Sigurdsson's previous work (Boutajangout et al., 2011) locomotor activity of the IgG-treated mice showed “distance traveled” of ~7,800 cm per mouse on average over 15 min. The same test, in the current paper, showed ~3,000 cm for IgG-treated (control treatment) mice—less than half of the previously reported value. The authors also reported that 27% of the mice in their current study (4 control mice and 2 treated mice) died during the experiment, which strongly indicates that the animals were at a much more advanced stage of the disease than that previously tested, with a severe motor deficit. Therefore, the current results cannot be interpreted without a positive-control, e.g., using the authors tau immunization approach as in Sigurdsson's previous works (Asuni et al., 2007; Boutajangout et al., 2011) to verify feasibility of detecting any treatment response in this “shifted” colony. In addition, age-matched healthy control mice are missing, as historical controls are meaningless in behavioral measures.

Independently of the above critical issues, the regimen of weekly treatment for “chronic PD-1/PD-L1 immune checkpoint blockade,” has not only never been suggested as a therapeutic protocol for achieving long-term effects in Alzheimer's disease, but is in contrast to previous studies using PD-1 or PD-L1 blocking antibodies (Baruch et al., 2016; Rosenzweig et al., 2019). Specifically, it was shown that a single treatment with PD-1/PD-L1 blocking antibody is sufficient to mitigate cognitive decline and reduce brain pathology, and that chronic beneficial effect on cognitive performance over 12 weeks was achieved by 3 monthly injections of anti-PD-1 antibody in 5XFAD mice (Rosenzweig et al., 2019). In line with these results, ImmunoBrain Checkpoint Ltd. tested the effect of anti-PD-L1 antibody administration on cognitive performance in the double mutant tauopathy mouse model (K257T/P301S; double mutant, DM-hTAU), and found that a chronic beneficial effect could be maintained over a period of 4 months by injections every 6 weeks (Figure 1). Thus, for a chronic course of treatment, intermittent blockade is needed, where each treatment session includes a period of immune checkpoint blockade followed by a period free of antibody exposure. The issue of intermittent rather than continuous exposure was discussed in the two papers cited above, as well as in an Opinion article by Schwartz (2017).

**Figure 1:**
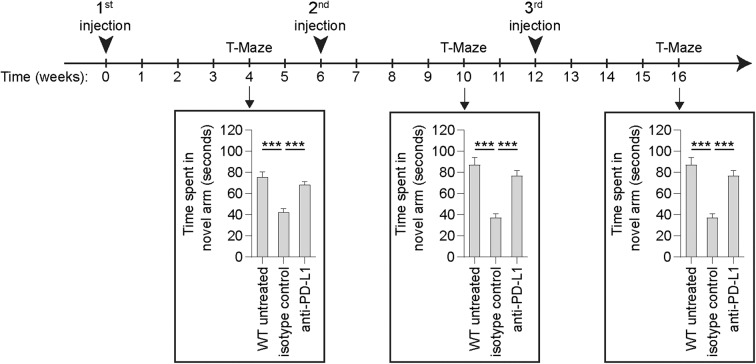
Longitudinal assessment of cognitive performance of DM-hTAU mice following anti-PD-L1 intermittent treatment regimen. Male and female DM-hTAU mice at the age of 6–7 months were treated by intraperitoneal injection of either 1.5 mg/mouse of anti-PD-L1 antibody, or 1.5 mg/mouse isotype control antibody, once every 6 weeks. Untreated age-matched wild-type (WT) mice were used as an additional control group. Using the same protocols described in Rosenzweig et al. (2019), mice were evaluated for the effect on cognitive performance using the T-maze task, 4 weeks after each injection. Preference to spend time in the novel arm of the maze is a measure of short-term spatial memory. *n* = 54 DM-hTAU mice and *n* = 13 WT mice for the T-maze at 4 weeks from treatment initiation; *n* = 48 DM-hTAU mice and *n* = 21 WT mice for the T-maze at 10 weeks from treatment initiation; and *n* = 30 DM-hTAU mice and *n* = 15 WT mice for the T-maze at 16 weeks from treatment initiation. One-way ANOVA followed by Fisher's *post-hoc* test. Error bars represent mean ± s.e.m.; ***P < 0.001 vs. indicated groups. Mice were sacrificed along study progression for additional measurements, not presented here [ImmunoBrain Checkpoint Ltd.].

Critically, the justification by Lin et al. for the selected weekly injections of anti-PD-1 antibody is based on their regimen for tau antibody therapy. Such justification ignores the fact that choice of regimen for any antibody therapy must be based on its mechanism of action. There is no scientific or therapeutic basis to justify any mechanistic linkage between anti-amyloid/tau antibody approaches employed in Alzheimer's disease, and the use of anti-PD-1/PD-L1 antibodies, which represent a completely different mechanism of action of the therapeutic approach. While amyloid and tau antibodies are designed to directly dampen the pathology within the brain, PD-1/PD-L1 antibodies are targeting immune cells outside the brain. Thus, PD-1/PD-L1 blockade in mouse models of Alzheimer's disease initiates a chain of immunological events that start in the periphery and culminate within the brain's territory; beginning with the antibody recognizing its cellular targets in the periphery and transiently breaking immune tolerance, and this is followed by migration of specialized immune cell populations from the circulation to the brain (thoroughly described in: Baruch et al., 2016; Schwartz, 2017; Rosenzweig et al., 2019). Immune cells (primarily of myeloid origin) that are recruited to the brain, act by enhancing clearance of toxic elements, improving neuronal function and reducing inflammation. This central effect, within the brain's territory, does not require the presence of the PD-1/PD-L1 antibody, which by that time has been cleared from the circulation. Thus, as opposed to the concept of maintaining continuous exposure with amyloid/tau antibodies for chronic effect on brain pathology, for immune checkpoint blockade, injections should be given intermittently to maintain a chronic beneficial effect. Indeed, in Rosenzweig et al., it was stated that “…*the beneficial effect of the immunotherapy for AD and dementia does not require continuous exposure to the antibody, and that the effect is mechanistically different from that underlying the current anti-PD-L1 treatment used in cancer therapy*” (Rosenzweig et al., 2019).

In summary, Lin et al. performed an experiment missing key appropriate control groups, using a cohort of aged “shifted” transgenic mice, which exhibit a clear motor deficit, and for which no behavioral or pathological data are available. The anti-PD-1-based therapy was used in a regimen that lacks scientific basis, and contradicts the previously available literature describing the dynamics of the therapy. These deficiencies preclude reaching any conclusion from this work, and as such only contribute to the confusion in the field.

## Ethics Statement

Animal experiments detailed herein complied with the regulations formulated by the Institutional Animal Care and Use Committee (IACUC) of the Weizmann Institute of Science, Israel.

## Author Contributions

KB and EY conceived and wrote this commentary.

## Conflict of Interest

KB and EY work at ImmunoBrain Checkpoint Ltd., on the development of PD-1/PD-L1 immune checkpoint blockade approach for Alzheimer's disease. KB is an inventor of intellectual property licensed by ImmunoBrain Checkpoint Ltd.
